# Rab32‐related antimicrobial pathway is involved in the progression of dextran sodium sulfate‐induced colitis

**DOI:** 10.1002/2211-5463.12514

**Published:** 2018-09-21

**Authors:** Xiaodong Xie, Qingshan Ni, Daxue Zhou, Ying Wan

**Affiliations:** ^1^ Biomedical Analysis Center Army Medical University Chongqing China; ^2^ Chongqing Key Laboratory of Cytomics China

**Keywords:** bacteria, CD11c^+^ cells, colitis, inflammation, inflammatory bowel disease, Rab32

## Abstract

Inflammatory bowel disease (IBD) is a multifactorial disease involving defective immune responses against invasive microbiota. Genes associated with innate immune responses to microbes have been highlighted in the pathogenesis of IBD. To determine the role of Rab32 in the pathogenesis of IBD, we administered dextran sodium sulfate (DSS) to CD11c^+^ cell‐specific Rab32 knockout (*CD11c*‐Cre^+^Rab32^f/f^) mice to induce colitis. Rab32 deficiency in CD11c^+^ cells resulted in more severe disease progression and increased mortality. Histopathological analysis showed extensive damage to the colon mucosa in DSS‐treated *CD11c*‐Cre^+^Rab32^f/f^ mice, including more severe damage to the epithelial layer and crypts, as well as more inflammatory cell infiltration. The pro‐inflammatory cytokines IL1A, IL1B, IL6, and CSF3 and chemokines CXCL1 and CXCL2 were significantly increased, and the frequency of CD11b^+^Ly6G^+^ neutrophils was higher in *CD11c*‐Cre^+^Rab32^f/f^ colitis mice. Furthermore, CD11c^+^ cells deficient for Rab32 exhibited a significant increase in bacterial translocation in inflamed colon tissue. The present data demonstrate that Rab32 knockout in CD11c^+^ cells aggravates the development of DSS‐induced colitis and suggest that the Rab32‐related antimicrobial pathway is involved in the pathogenesis of IBD.

**Abbreviations**
BMDC: bone marrow‐derived dendritic cell
BMDM: bone marrow‐derived macrophage
CD: Crohn's disease
CFU: colony‐forming unit
DC: dendritic cell
DSS: dextran sodium sulfate
IBD: inflammatory bowel disease
IEL: intraepithelial lymphocyte
LPL: lamina propria lymphocyte
MLN: mesenteric lymphoid node
MP: macrophage
rDNA: ribosome DNA

Inflammatory bowel disease (IBD) is a prevalent chronic inflammatory disorder in the digestive tract that comprises two main types, Crohn's disease (CD) and ulcerative colitis. Abdominal pain, diarrhoea and rectal bleeding are common symptoms of IBD patients 1, 2. Since the 1950s, the incidence of IBD has increased, and currently, the prevalence of this disease is up to 0.5% of the general population in Western countries 3, 4. The pathogenesis of IBD is multifactorial, including environmental factors, genetic susceptibility, dysregulated immunity and infectious microbes 5. Genomic studies have identified more than 200 loci containing IBD susceptibility genes, focusing on critical pathways involved in innate immunity, autophagy, lymphocyte differentiation and chemotaxis 6, 7.

Among these loci, a number of genes associated with microbial sensing (NOD2, REL, IRF5, RELA, PTPN22, CARD9 and RIPK2) and clearance (ATG16L1, NCF4I and RGM) have been highlighted 8, 9, 10, 11. In the gastrointestinal tract lamina propria, the intestinal dendritic cells (DCs) and macrophages (MPs) provide first‐line defence against invading microorganisms 12. Disruptions in the DCs and MPs lead to bacterial translocation across the intestinal mucosa and subsequent inflammation 13. Accumulated evidence in IBD patients has demonstrated that dysfunctional bacteria clearance ability increases bacterial invasion in mucosa biopsies 14, 15. However, more mechanisms underlying the innate immune response against microbial invasion during IBD progression remain unexplored.

Recently, Rab32 was identified as an essential player in the clearance of intracellular pathogens both in DCs and MPs 16, 17, 18. Moreover, Rab32 has been found to be frequently methylated in IBD and IBD‐related neoplasms 19, 20. In this study, we examined the role of Rab32 in a dextran sodium sulfate (DSS)‐induced colitis model. We found that Rab32 deficiency in CD11c^+^ cells exacerbates the progression of colitis in mice with increased neutrophil infiltration and bacterial invasion.

## Materials and methods

### Mice

*Rab32* ^*tm1a(KOMP)Wtsi*^ mice were purchased from the KOMP Repository, and CD11c‐Cre (B6.Cg‐Tg^(Itgax−cre)1−1Reiz/J^) and Flpe mice (129S4/SvJaeSor‐Gt(ROSA)26Sor^tm1(FLP1)Dym/J^) mice were purchased from the Jackson Laboratory. CD11c‐specific Rab32‐deficient mice (*CD11c*‐Cre^+^Rab32^f/f^) were generated according to our published paper 16. *Rab32* ^*tm1a(KOMP)Wtsi*^ mice were initially crossed with Flpe mice and then crossed with CD11c‐Cre mice to delete the second exon of Rab32. All of the mice were bred under specific‐pathogen‐free conditions. All animal experiments were conducted on protocols that were approved by the Medicine Animal Care Committee of the Army Medical University (Chongqing, China).

### Bone marrow‐derived dendritic cell and macrophage culture

Bone marrow‐derived dendritic cells (BMDCs) and bone marrow‐derived macrophages (BMDMs) were generated according to published protocols 21, 22. To generate BMDCs, we cultured bone marrow isolated from tibiae and femora in 24‐well plates with 500 μL medium per well [RPMI 1640 containing 10% FBS, 2 mmol·L^−1^ l‐glutamine, 50 μmol·L^−1^ 2‐mercaptoethanol, 200 000 U·L^−1^ penicillin, 200 000 U·L^−1^ streptomycin and 20 ng·mL GM‐CSF (Peprotech, Rocky Hill, NJ, USA)]. On day 2, the medium was removed and replaced with 37 °C prewarmed fresh medium. On day 4 and day 6, half of the medium was replaced with fresh prewarmed medium. On day 7, nonadherent and loosely adherent cells were harvested. To generated BMDMs, the isolated bone marrow was cultured in a 10‐cm dish with 10 mL medium [RPMI 1640 containing 10% FBS, 2 mmol·L^−1^ l‐glutamine, 50 μmol·L^−1^ 2‐mercaptoethanol, 200 000 U·L^−1^ penicillin, 200 000 U·L^−1^ streptomycin and 20 ng·mL^−1^ M‐CSF (Peprotech)]. On day 3 and day 6, half of the medium was replaced with 37 °C prewarmed fresh medium containing 40 ng·mL^−1^ M‐CSF. On day 7, the adherent cells were collected. The CD11c^+^ cells were sorted for purified BMDCs, and CD11b^+^F4/80^+^ cells were sorted for purified BMDMs.

### Western blot

For western blot experiments, 1 × 10^6^ harvested cells were lysed on ice for 30 min in 50 μL of lysis buffer consisting of protease inhibitor cocktail (Roche, Basel, Switzerland). After centrifugation at 10 000 ***g*** for 10 min at 4 °C, supernatants were collected. The concentrations of total protein were determined using the BCA (Beyotime, Shanghai, China) method. Denatured cellular protein lysates were separated in 4%–12% gradient NuPAGE^®^ Novex Bis‐Tris gels (Invitrogen, Carlsbad, CA, USA) and transferred to PVDF membranes (Millipore, Bedford, MA, USA). The membranes were subsequently blocked in PBS containing 0.05% Tween and 5% nonfat milk (Bio‐Rad, Hercules, CA, USA) overnight at 4 °C and then incubated for 1 h with the primary antibody goat anti‐mouse Rab32 (1 : 1000; Abcam, Cambridge, UK) or rabbit anti‐mouse β‐actin or β‐tubulin (1 : 1000; Abcam). The membranes were washed with PBST 3 times for 10 min and then incubated with HRP‐conjugated anti‐goat IgG (1 : 1000; CST, Boston, MA, USA) and anti‐rabbit IgG (1 : 1000; CST) secondary antibodies. Signals were visualised by chemiluminescence using the ChemiDoc Touch Imaging system (Bio‐Rad).

### Induction of colitis and evaluation of colitis severity

Mice (8‐ to 12‐week‐old) received 2.5% DSS (MW36‐50 kDa, MP Biomedicals, USA) in their drinking water for 8 days. DSS‐induced colitis severity was scored daily by evaluating disease activity index (DAI) as reported 23, 24, 25 with minor modification. The DAI score was calculated as the sum of a four‐item score, including weight loss (0 points = no change, 1 point = < 5%, 2 points = 6–10%, 3 points = 11–20% and 4 points = more than 21%), general appearance (0 points = normal, 1 point = piloerection, 2 points = lethargy and piloerection and 4 points = motionless and sickly), faeces consistency (0 points = normal, 2 points = pasty and semiformed and 4 points = liquid, sticky or unable to defecate after 5 min) and rectal bleeding (0 points = no blood, 2 points = visible blood in the rectum and 4 points = visible blood on the fur).

### Histological analysis

The caecum and colon were fixed with paraformaldehyde and stained with haematoxylin and eosin (HE). The colonic inflammation degree was evaluated based on a previously validated scoring system 25, 26, 27 as follows: infiltration (0 points = rare infiltration, 1 point = increased number of inflammatory cells in the mucosa and 2 points = confluence of inflammatory cells extending into the submucosa), crypt damage (0 points = none, 1 point = basal 1/3 damage, 2 points = basal 2/3 damage, 3 points = only surface epithelium intact and 4 points = entire crypt and epithelium damage) and percentage of area involved (0 points = none, 1 point = 1–25%, 2 points = 26–50%, 3 points = 51–75% and 4 points = 76–100%). The colon was equally divided into four segments. The proximal colon score was determined as the average of the upper two segments, and the distal colon score was calculated as the average of the lower two segments.

### Cell isolation and flow cytometry analysis

Intraepithelial (IEL) and lamina propria lymphocytes (LPL) were isolated from the colon as previously described 28. Briefly, the colon was flushed with 5 mL of PBS to remove the contents; then, it was opened longitudinally and cut into ~ 0.5‐cm pieces. To harvest IEL, the tissues were incubated twice with 5 mm EDTA containing 2% FBS at 37 °C for 20 min and the supernatant was collected. To isolate LPL, the tissues were digested twice in 0.5 mg collagenase D (Roche)/100 mL RPMI 1640 containing 5% FBS at 37 °C for 30 min and the supernatant was subsequently collected. 40%/80% Percoll (GE Healthcare, Uppsala, Sweden) step‐gradient was used to enrich IEL and LPL. Mesenteric lymphoid nodes (MLNs) were mechanically dispersed through a stainless steel 200‐μm wire mesh in PBS, and the cells were washed after centrifugation for 5 min at 500 g. The cells were incubated with FITC‐conjugated CD3, PE‐conjugated CD4, PerCP‐cy5.5‐conjugated CD8a, APC‐cy7‐conjugated CD11b and APC‐conjugated Ly6G (all antibodies from eBioscience, San Diego, CA, USA) antibodies at 4 °C for 30 min, and DAPI was used to stain for dead cells at 4 °C for 5 min. The cells were subsequently detected on the BD FACSVerse platform and analysed with flowjov10 software (San Carlos, CA, USA).

### Real‐time qPCR

RNA was extracted from the colon tissues using an RNeasy mini kit (Qiagen, Hilden, Germany) according to the manufacturer's instructions. Total RNA (1 μg) was reverse‐transcribed into cDNA with a reverse transcription kit (Sigma, St. Louis, MO, USA) and quantified with SYBR Green‐based real‐time qPCR with specific primers for IL1A, IL1B, IL6, IL 17, IFNγ and TNFα (synthesised at BGI, China). The inflammatory‐related genes were summarised from the published studies. All the designed primers for these genes (135 genes) were optimised and organised into the inflammatory response qPCR array primer plate (Table S1). The qPCR array reaction was performed in a 384‐well optical plate with a 2.0 μL total reaction volume by liquid handling with Echo 550 (Labycte, Sunnyvale, CA, USA).

A16S ribosome DNA (rDNA) qPCR was used to quantify the bacteria of the colon, MLNs and spleen as previously described 29. Briefly, the spleen, MLN and colon were separated, and the colon was flushed with 5 mL of sterile PBS to remove the contents. DNA was extracted using a DNA isolation kit (Qiagen) and amplified using primer sets for 16S rDNA of universal bacteria. The sequences of the primers used for real‐time qPCR are shown in Table S2.

The CT value of all the qPCR products was normalised to housekeeping genes, and the quantitated relative expression levels were determined using ΔΔC_T_ analysis. In the qPCR array analysis, the limma package in R was used to identify genes that were differentially expressed between experimental conditions 30.

### Bacterial culture

The colon, MLNs and spleen were separated from DSS‐induced colitis mice on day 8. The colon was flushed with 5 mL of sterile PBS to remove the colon contents. Then, the colon tissues, MLN and spleen were homogenised in sterile PBS and plated onto BBL Brucella agar plates (containing 5% horse blood) under sterile conditions as previously described 29. The plates were incubated for 48 h at 37 °C under anaerobic conditions, followed by colony‐forming unit (CFU) counting.

### Statistical analysis

Statistical analysis was performed by using graphpad prism 6 software (Graphpad software Inc., San Diego, CA, USA) to calculate unpaired Student's *t*‐test or *Log‐rank* test. All *P*‐values < 0.05 were considered to indicate significant results and were marked with asterisk (*P < 0.05; **P < 0.01; ***P < 0.001).

## Results

### Rab32 knockout in CD11c^+^ cells aggravates the development of DSS‐induced colitis

**Figure 1:**
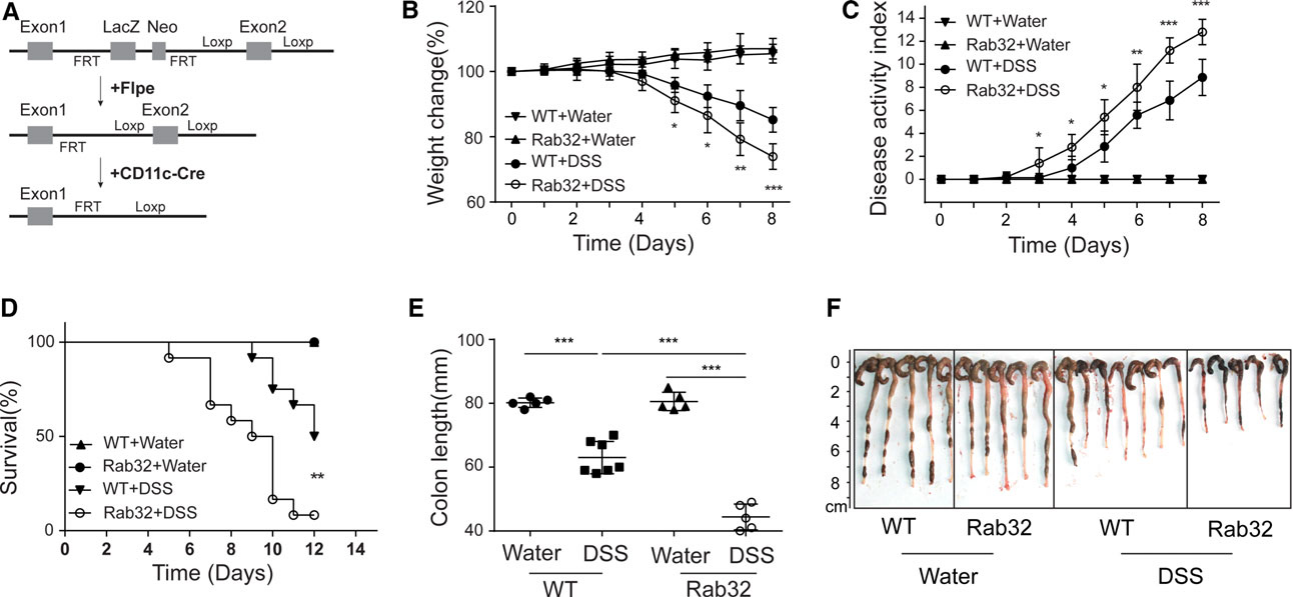
Rab32 deficiency in CD11c^+^ cells exacerbates the progression of DSS‐induced colitis. (A) Schematic diagram of CD11c‐*Cre* ^*+*^ *Rab32* ^*f/f*^ mice generation. (B, C) Body weight change and disease index activity were monitored daily (*n* = 5–8 mice/group). Colitis was induced by the oral administration of 2.5% DSS. Mice orally administered normal distilled water were used as controls. (D) The survival rate of CD11c*‐Cre* ^*+*^ *Rab32* ^*f/f*^ mice and WT mice treated with DSS or water (*n* = 10 mice/group). The survival of mice was recorded daily, and the survival rate was calculated using the Kaplan–Meier test. (E, F) Length and gross imaging of colons dissected from the *CD11c*‐Cre^+^Rab32^f/f^ and WT mice administered with DSS or water. All data are shown as the mean ± SD. Significances were calculated using unpaired Student's *t*‐test or *Log‐rank* test. *P < 0.05, **P < 0.01 and ***P < 0.001.

To investigate the roles of Rab32 in CD11c^+^ cells in DSS‐induced colitis, we generated CD11c^+^ cell‐specific Rab32‐deficient (*CD11c*‐Cre^+^Rab32^f/f^) mice (Fig. 1A). Deletion of Rab32 in CD11c^+^ cells was confirmed by PCR and western blot analysis of Rab32 in BMDCs at the genomic and protein levels (Fig. S1). DSS‐induced colitis is a classical disease model with many similarities to the clinical manifestations of colitis in human IBD patients. After orally administering 2.5% DSS in drinking water for 8 days, DSS‐induced weight loss was significantly higher in *CD11c*‐Cre^+^Rab32^f/f^ mice than in WT mice (Fig. 1B). *CD11c*‐Cre^+^Rab32^f/f^ mice weighed 73.9 ± 3.5% of their original body weight, while WT mice weighed 85.2 ± 3.5% of their original body weight. To more comprehensively evaluate the disease progression, we monitored mice daily with a disease activity index (DAI) that includes weight loss, general appearance, diarrhoea and rectal bleeding. The weight loss in *CD11c*‐Cre^+^Rab32^f/f^ mice began on day 4, and diarrhoea with bleeding was clearly detected on day 7, while weight loss began on day 5 in the WT mice, and little bleeding was observed. The DAI score of *CD11c*‐Cre^+^Rab32^f/f^ mice was significantly higher than that of control mice (*P* < 0.001, Fig. 1C). Moreover, the mortality of the *CD11c*‐Cre^+^Rab32^f/f^ mice reached over 90% when post continuously treated with 2.5% DSS but reached only approximately 50% in the WT mice (Fig. 1D). These data demonstrate that Rab32 deficiency in CD11c^+^ cells results in more severe disease progression and increased mortality.

**Figure 2:**
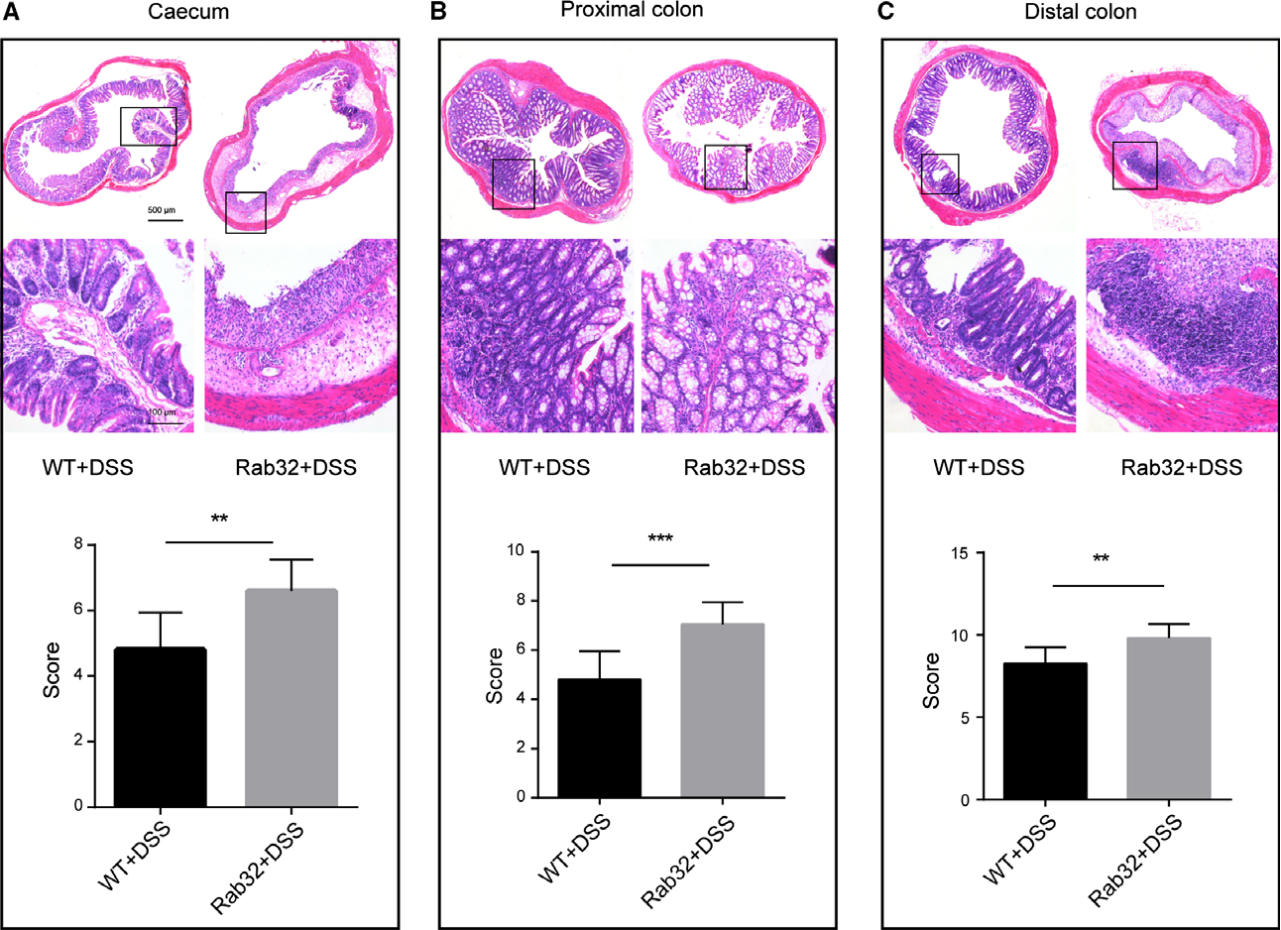
Histological analysis of inflamed tissue. HE‐stained sections of the (A) caecum, (B) proximal colon and (C) distal colon from WT and *CD11c‐*Cre^+^Rab32^f/f^ colitis mice were microscopically examined at 40X and 200X, and histology scores were analysed for 6–8 mice from each group. Scale bar: 500, 100 μm. All data are shown as the means ± SD. Significances were calculated using unpaired Student's *t*‐test. **P < 0.01 and ***P < 0.001.

Consistent with the clinical score data, the colon of *CD11c*‐Cre^+^Rab32^f/f^ mice was shorter than that of control mice (44.4 ± 3.6 mm versus 63.1 ± 4.7 mm, respectively, *P* < 0.001, Fig. 1E,F). Histological scores revealed a significantly greater degree of tissue injury in *CD11c*‐Cre^+^Rab32^f/f^ mice than in WT control mice after DSS treatment (Fig. 2), although no significant differences were observed between *CD11c*‐Cre^+^Rab32^f/f^ and WT mice administered water (Fig. S2).

### Increased pro‐inflammatory cytokine expression and neutrophil infiltration in the inflamed colon tissue of *CD11c*‐Cre^+^Rab32^f/f^ mice

**Figure 3:**
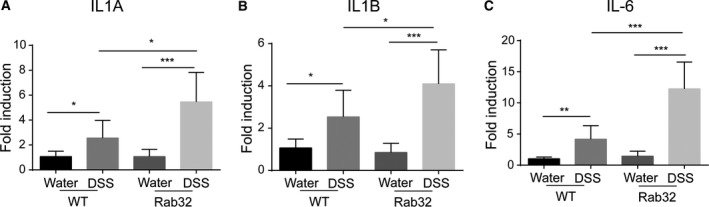
Aberrant expression of pro‐inflammatory cytokines in *CD11c*‐Cre^+^Rab32^f/f^ colitis mice. Total RNA was extracted from the colons tissues of untreated and DSS‐treated mice on day 8. Expression of the pro‐inflammatory cytokines (A) IL1α, (B) IL1β and (C) IL6 was measured by qPCR (*n* = 6–8 mice/group). All data are shown as the means ± SD. Significances were calculated using unpaired Student's *t*‐test. *P < 0.05, **P < 0.01 and ***P < 0.001.

**Figure 4:**
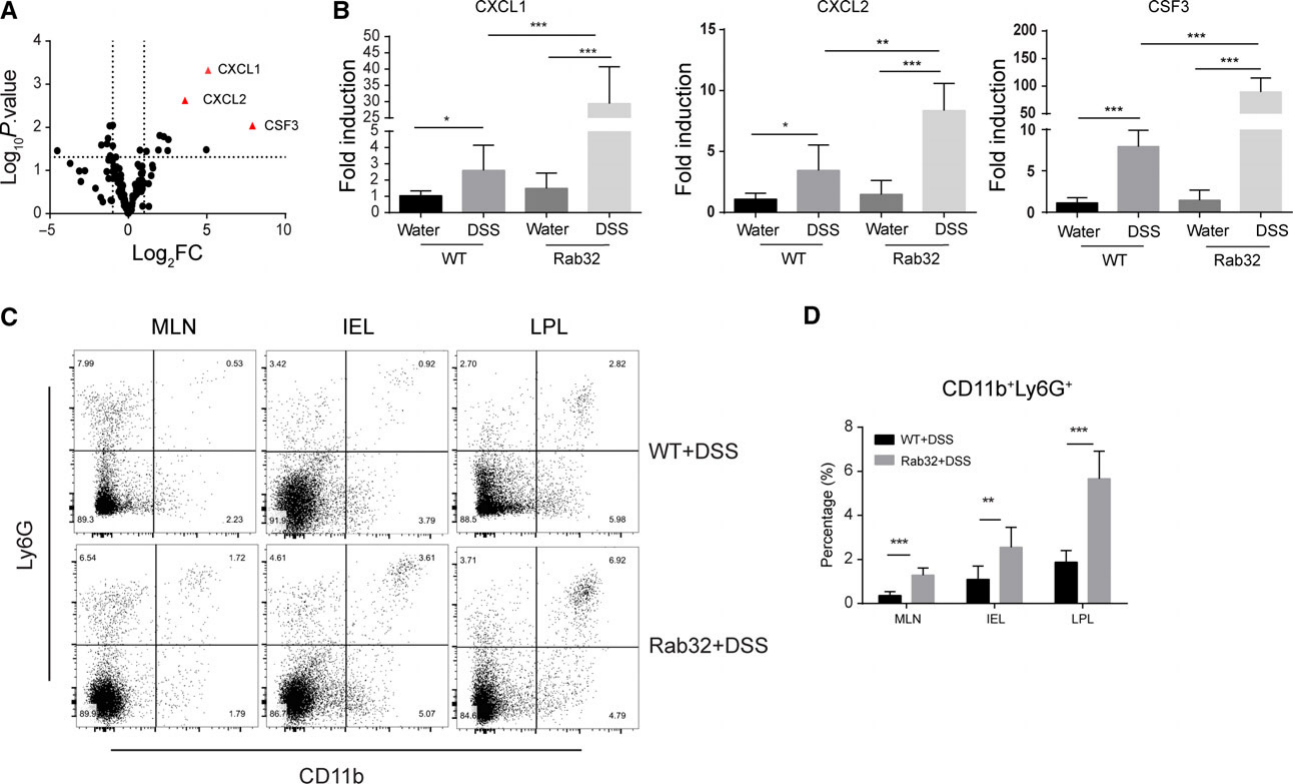
Increased chemokines expression and neutrophils infiltration in *CD11c*‐Cre^+^Rab32^f/f^ colitis mice. (A) Total RNA was extracted from the colon tissues of untreated and DSS‐treated mice to analyse the expression of 135 mouse inflammatory‐related genes with qPCR, 3 mice in each group. (B) The most significantly expressed genes CXCL1, CXCL2 and CSF3 were verified with qPCR, 6–8 mice in each group. (C, D) The frequencies of neutrophils (CD11b^+^Ly6G^+^) in isolated colonic IEL, LPL and MLNs from mice in the indicated groups were determined by FACS, 5–8 mice in each group. All data are shown as the means ± SD. Significances were calculated using unpaired Student's *t*‐test. *P < 0.05, **P < 0.01 and ***P < 0.001.

Cytokines, including interleukins and interferons, play a critical role in the pathogenesis of IBD and are dramatically upregulated in the DSS model 31. The inflammatory cytokines IL1A, IL1B, IL6, IL17, TNFα and IFNγ were investigated first. IL1A, IL1B and IL‐6 were significantly increased in *CD11c*‐Cre+Rab32^f/f^ mice (Fig. 3), while the cytokines IL17, TNFα and IFNγ showed no changes (Fig. S3). Using a homemade inflammatory response qPCR array, the expression of 135 inflammatory‐related genes, including cytokines, chemokines and chemokine receptors, and transcriptional factors was assessed (Table S1). The expression of chemokines CXCL1, CXCL2 and CSF3 was significantly increased in *CD11c*‐Cre+Rab32^f/f^ mice (Fig. 4A,B). However, the cytokines and transcription factors associated with the T‐cell response and differentiation, such as IL4, IL12, IL10, Granzyme A/B, STAT4, GATA3, RORC and FOXP3, showed no differences. These data suggest that the severe colonic inflammation in *CD11c*‐Cre^+^Rab32^f/f^ mice may not be caused by dysfunctional T cells.

Intraepithelial and LPL were isolated and analysed by FACS. The frequency of CD3^+^, CD4^+^ and CD8^+^ T cells showed no significant differences between the *CD11c*‐Cre^+^Rab32^f/f^ and WT mice (Fig. S3). The expression of CD80, CD86 and MHCII also showed no differences in CD11c^+^ BMDCs after LPS stimulation (Fig. S4). However, the frequency of CD11b^+^Ly6G^+^ neutrophils was higher in *CD11c*‐Cre^+^Rab32^f/f^ colitis mice than in the WT colitis mice (Fig. 4C,D). No significant differences were observed between *CD11c*‐Cre^+^Rab32^f/f^ and WT mice administered water (Fig. S5). The MP chemokines CXCL1/CXCL2 were reported to control the early stage of neutrophil recruitment during tissue inflammation 32, 33, 34. These data indicate that the severe tissue damage of *CD11c*‐Cre^+^Rab32^f/f^ colitis mice was related to the abundant neutrophil infiltration, which were recruited by the increased expression of chemokines in the tissues.

### *CD11c*‐Cre^+^Rab32^f/f^ mice are more susceptible to bacterial invasion

**Figure 5:**
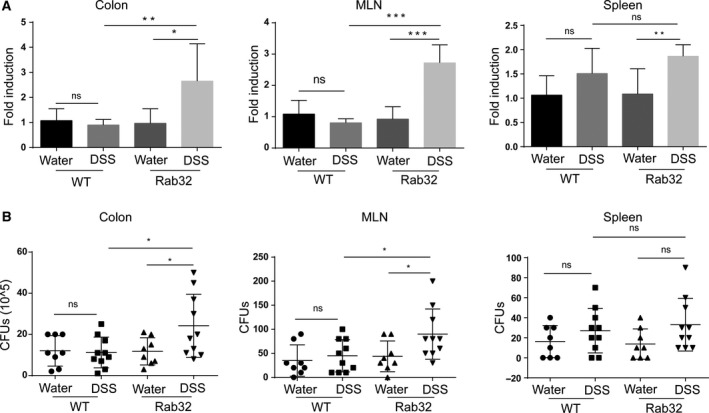
Increased bacterial invasion in *CD11c*‐Cre^+^Rab32^f/f^ colitis tissue. The spleen, MLN and colon were separated from untreated and DSS‐treated mice. The colon was flushed with sterile PBS to remove the contents. (A) The translocated bacteria in inflamed tissues were assessed with 16S rDNA quantitative PCR (*n* = 8–10 mice/group). (B) The colon tissues, MLN and spleen were homogenised and incubated onto BBL Brucella agar plates for 48 h at 37 °C under anaerobic conditions, followed by CFU counting (*n* = 8–10 mice/group). All data are shown as the means ± SD. Significances were calculated using unpaired Student's *t*‐test. *P < 0.05, **P < 0.01 and ***P < 0.001.

Neutrophils are the critical components in the innate immune response against the invasion microbes and can be recruited by bacteria‐derived products 35, 36. During DSS administration, the epithelial layer is destroyed, and bacteria infiltrate the intestinal mucosa 29. Therefore, we assessed the bacterial infiltration in the DSS colitis mice. By quantifying the bacterial 16S rDNA with real‐time qPCR, we found that *CD11c*‐Cre^+^Rab32^f/f^ and WT mice administered water showed no differences in the level of bacterial invasion. However, after DSS administration, bacterial 16S rDNA copies were significantly increased in the MLN and colons. Under anaerobic culture, the colon tissues and MLN of *CD11c*‐Cre^+^Rab32^f/f^ mice showed more bacterial growth. No significant differences were observed in the spleen between the two groups through qPCR of 16S rDNA and the anaerobic culture (Fig. 5A,B). These data indicated that commensal bacteria could be resisted in a healthy state of *CD11c*‐Cre^+^Rab32^f/f^ mice, but when the mucosal was damaged by DSS, the deficiency of Rab32 in CD11c^+^ cells causes more bacterial translocation in inflamed colon tissue and MLN.

## Discussion

Accumulating studies have highlighted the importance of innate immune responses to microbes in the pathogenesis of IBDs. Blood monocytes isolated from IBD patients showed decreased phagocytosis and killing of bacteria 37. Recent studies determined that MPs isolated from IBD patients showed decreased reactive oxygen species production 38, 39. Defects in autophagy impaired the killing of adherent‐invasive E. coli either in DCs or in MPs 11, 40. Deletion of autophagy‐related gene ATG16L1 in CD11c^+^ cells results in more severe DSS‐induced colitis in mice 41. In this study, we found that Rab32 deficiency in CD11c^+^ cells aggravates the progression of DSS‐induced colitis, with a more severe inflammation appearance, colonic tissue damages and higher mortalities. The pro‐inflammatory cytokines (IL1A, IL1B and IL‐6), neutrophils and bacterial infiltration were significantly increased in *CD11c*‐Cre^+^Rab32^f/f^ mice with colitis induced by DSS. These data suggest that the CD11c positive cells play an important role in the process of DSS‐induced colitis by defending the translocation of microbiota. Although the CD11c is a traditional surface marker to identify DCs, a subset of MPs in gastrointestinal tract express low to intermediate amounts of CD11c 42. Both types of CD11c positive cells are known to have bacterial processing capability. We do not rule out which subset of CD11c^+^ cells with a deletion in Rab32 contribute to the effects we observed. Future studies using deletions placed behind alternative promoters may clarify the picture.

Massive amounts of neutrophils were recruited to the colon tissues of IBD patients 43. Although the specific role of neutrophils during colitis has not been clearly delineated, some reports suggest that neutrophils are rapid responders to eliminate the translocated microbes across the intestinal epithelial layer 44. Our data support the hypothesis because Rab32 deficiency in CD11c^+^ cells resulted in a more severe colitis progression and increased the frequency of CD11b^+^Ly6G^+^ neutrophils in inflamed tissue. It has been reported that MP chemokines CXCL1/CXCL2 contribute to the recruitment of neutrophils at an early stage of tissue inflammation 45. In this study, pro‐inflammatory cytokines IL1A, IL1B, IL6, and CSF3 and chemokines CXCL1 and CXCL2 were significantly increased in *CD11c*‐Cre^+^Rab32^f/f^ mice. It is possible that the deficiency of Rab32 in CD11c^+^ cells recruits more neutrophils by chemokines and growth factors to cause more serious tissue damage 46, 47.

Rab32 has been well characterised in intracellular vesicle trafficking processes involving lysosome‐related organelle biogenesis, apoptosis and autophagy 48, 49, 50. Recent studies have demonstrated the critical role of Rab32 in bacteria restriction and infectious diseases 51, 52. Genome‐wide association studies identified IL23R and Rab32 as new susceptibility genes of leprosy, a disease caused by *Mycobacterium leprae* 53. Rab32 and its guanine nucleotide exchange factor BLOC‐3 are essential to prevent the growth of the human‐restricted *salmonella enterica serovar Typhi* (*S. Typhi)* in mice 17. In our previous study, Rab32‐Phb‐Phb2 complex‐associated membrane recaptures escaped *Listeria* to control *Listeria* replication in cytosol 16. Here, the deficiency of Rab32 in CD11c^+^ cells significantly increased the bacterial translocation in inflamed colon tissue after the mucosa was damaged by DSS. The data indicate the Rab32‐related antimicrobial pathway is involved in the progress of colitis.

In summary, the present data demonstrated that Rab32 in CD11c^+^ cells protects against the development of DSS‐induced colitis by preventing bacterial invasion and suggested that Rab32 might contribute to the pathogenesis of human IBD and IBD‐related colorectal carcinoma.

## Author contributions

performed experiments, analysed data and drafted paper. QN analysed data. DZ performed experiments. YW designed experiments and revised the manuscript.

## Accession numbers

The inflammatory‐related qPCR array was deposited in the Gene Expression Omnibus under the accession number GSE115035.

## Supporting information


**Fig. S1.** Analysis of the *CD11c‐*Cre^+^Rab32^f/f^ mice. BMDCs and BMDMs generated from WT and *CD11c‐*Cre^+^Rab32^f/f^ mice. (A) PCR analysis with primers amplified from exon1 and exon 3 of Rab32 with genomic from BMDCs. (B) The expression of Rab32 protein in BMDCs generated from WT and *CD11c‐*Cre^+^Rab32^f/f^ mice were analysed by Western blot. (C) The expression of Rab32 protein in BMDMs of WT and *CD11c‐*Cre^+^Rab32^f/f^ mice were analysed by Western blot.

**Fig. S2.** Histology analysis of colon tissue in the WT and *CD11c*‐Cre^+^Rab32^f/f^ mice administered water. HE‐stained sections of the (A) caecum, (B) proximal colon and (C) distal colon from WT and *CD11c‐*Cre^+^Rab32^f/f^ mice were microscopically examined at 40X and 200X, and histology scores were analysed for 7–8 mice each group. Scale bar: 500, 100 μm. All data are shown as the means ± SD.

**Fig. S3.** The expression of pro‐inflammatory cytokines and frequencies of T cells in the colon tissue. (A) Total RNA was extracted from the colons tissues of untreated and DSS treat mice to analyse the expression of pro‐inflammatory cytokines IL17, IFNγ and TNFα with qPCR. (B) The frequencies of CD3^+^, CD4^+^ and CD8^+^ T cells in the isolated colon from mice in the indicated groups were determined by FACS (*n* = 3 mice/group). All data are shown as the means ± SD.

**Fig. S4.** The maturation of BMDCs generated from WT and *CD11c*‐Cre^+^Rab32^f/f^ mice after stimulated with LPS. On Day 7 in culture, BMDCs were stimulated with 1 μg·mL^−1^ LPS for 24 h. (A) The surface marker MHC II molecule, CD80 and CD86 were analysed by FACS. (B) Proportions of MHC II^hi^, CD80^+^, and CD86^+^ BMDCs were calculated. The experiments were repeated 3 times. All data are shown as the means ± SD.

**Fig. S5.** The proportion of neutrophils infiltrated in the colon of the WT and *CD11c*‐Cre^+^Rab32^f/f^ mice administered water. The frequencies of neutrophils (CD11b^+^Ly6G^+^) in isolated colonic IEL, LPL and MLNs from mice in the indicated groups were determined by FACS (n = 5‐8 mice/group). All data are shown as the means ± SD.

**Table S1.** The primers of the inflammatory response qPCR array.

**Table S2.** Sequences of primers used for qPCR amplification.
