# Supplementary figures and images for: Rab32‐related antimicrobial pathway is involved in the progression of dextran sodium sulfate‐induced colitis
Source: FEBS Open Bio. 2018 Sep 21;8(10):1658–68. doi: 10.1002/2211-5463.12514 (PMC6168699; doi:10.1002/2211-5463.12514)

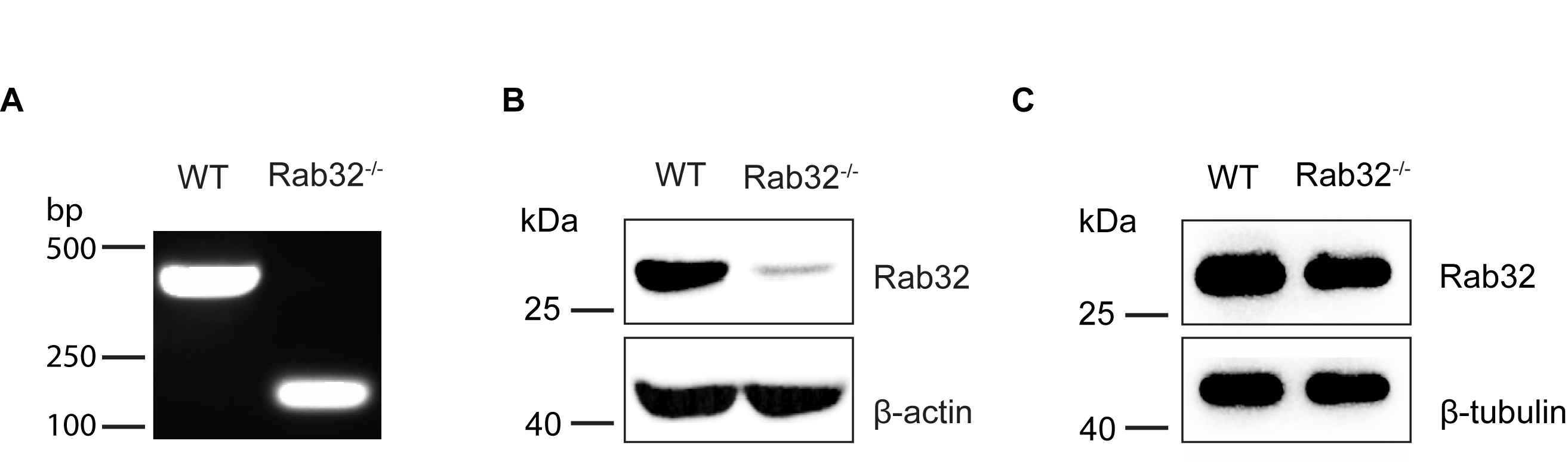

Supplement: Supplementary file 1 — Fig. S1. Analysis of the CD11c‐Cre+Rab32f/f mice. BMDCs and BMDMs generated from WT and CD11c‐Cre+Rab32f/f mice. (A) PCR analysis with primers amplified from exon1 and exon 3 of Rab32 with genomic from BMDCs. (B) The expression of Rab32 protein in BMDCs generated from WT and CD11c‐Cre+Rab32f/f mice were analysed by Western blot. (C) The expression of Rab32 protein in BMDMs of WT and CD11c‐Cre+Rab32f/f mice were analysed by Western blot. [file FEB4-8-1658-s001.tif]

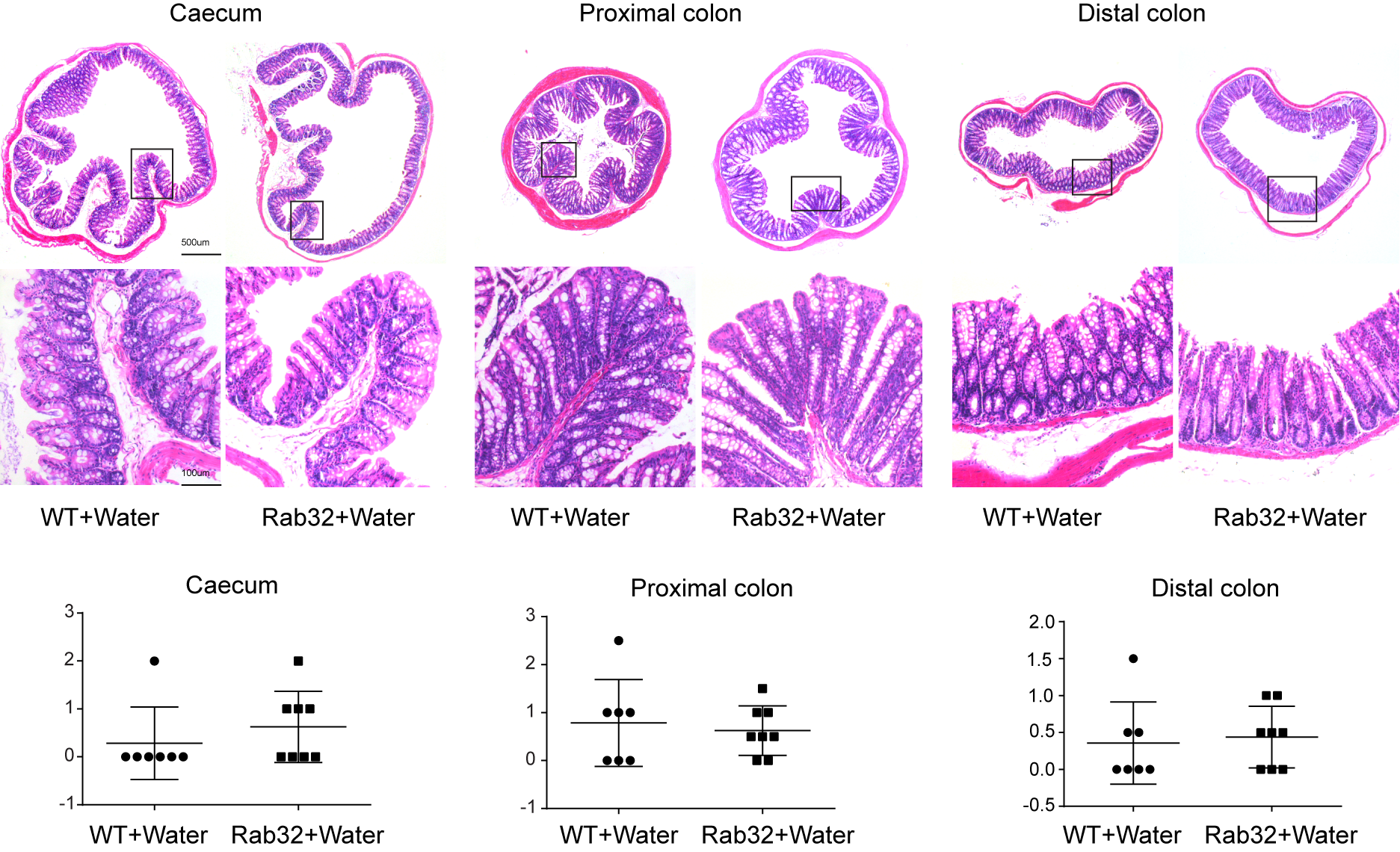

Supplement: Supplementary file 2 — Fig. S2. Histology analysis of colon tissue in the WT and CD11c‐Cre+Rab32f/f mice administered water. HE‐stained sections of the (A) caecum, (B) proximal colon and (C) distal colon from WT and CD11c‐Cre+Rab32f/f mice were microscopically examined at 40X and 200X, and histology scores were analysed for 7–8 mice each group. Scale bar: 500, 100 μm. All data are shown as the means ± SD. [file FEB4-8-1658-s002.tif]

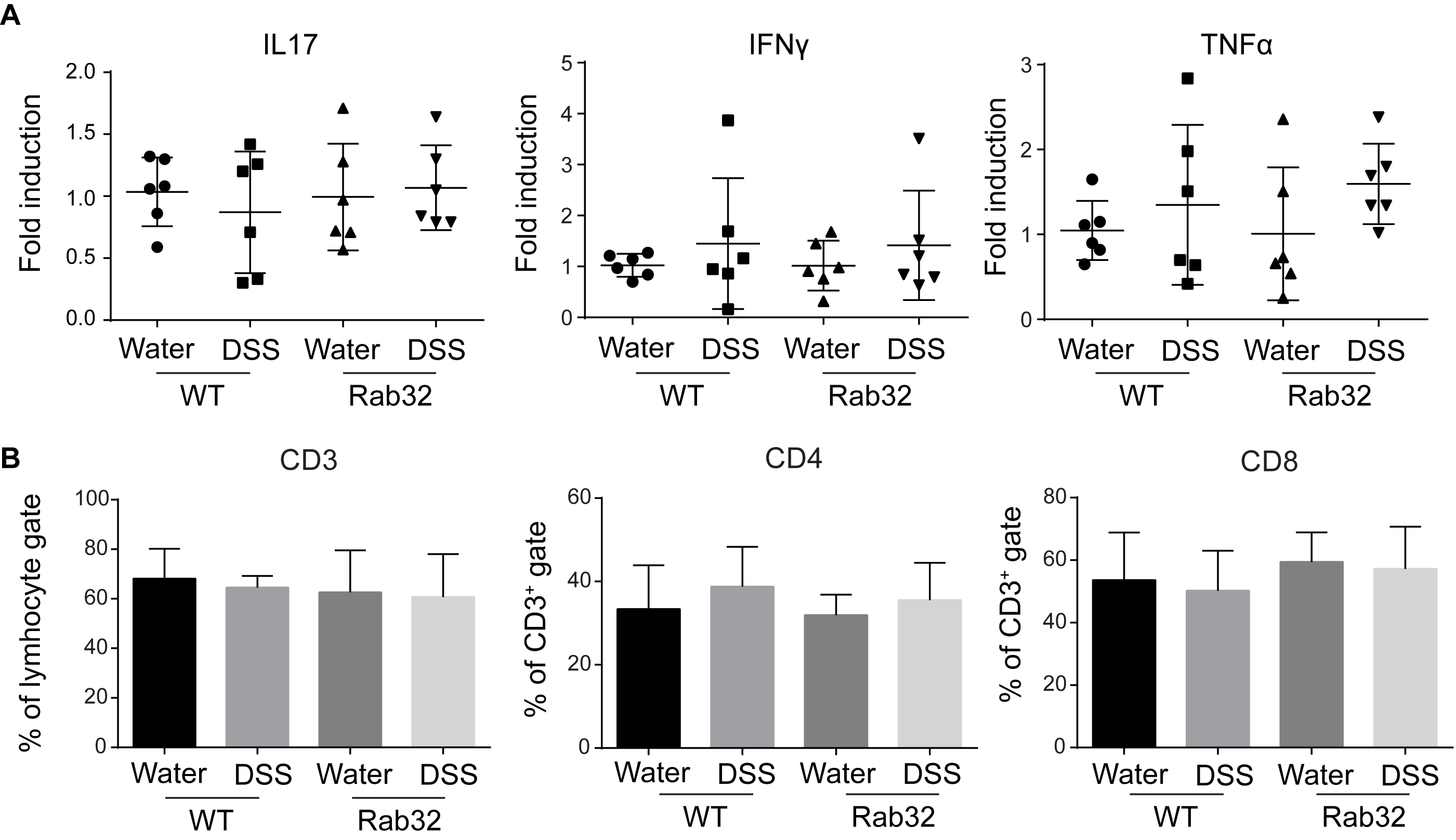

Supplement: Supplementary file 3 — Fig. S3. The expression of pro‐inflammatory cytokines and frequencies of T cells in the colon tissue. (A) Total RNA was extracted from the colons tissues of untreated and DSS treat mice to analyse the expression of pro‐inflammatory cytokines IL17, IFNγ and TNFα with qPCR. (B) The frequencies of CD3+, CD4+ and CD8+ T cells in the isolated colon from mice in the indicated groups were determined by FACS (n = 3 mice/group). All data are shown as the means ± SD. [file FEB4-8-1658-s003.tif]

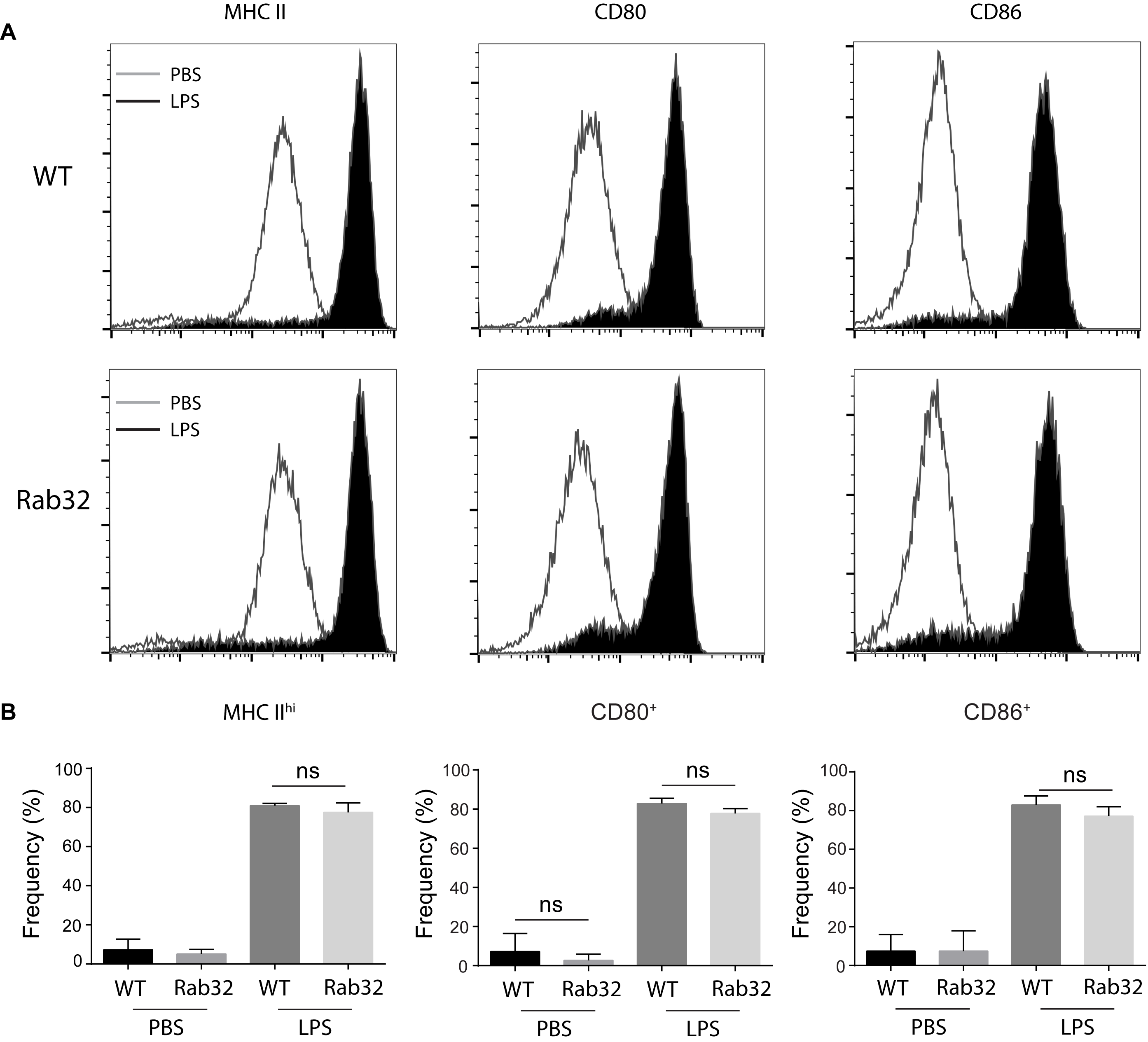

Supplement: Supplementary file 4 — Fig. S4. The maturation of BMDCs generated from WT and CD11c‐Cre+Rab32f/f mice after stimulated with LPS. On Day 7 in culture, BMDCs were stimulated with 1 μg·mL−1 LPS for 24 h. (A) The surface marker MHC II molecule, CD80 and CD86 were analysed by FACS. (B) Proportions of MHC IIhi, CD80+, and CD86+ BMDCs were calculated. The experiments were repeated 3 times. All data are shown as the means ± SD. [file FEB4-8-1658-s004.tif]

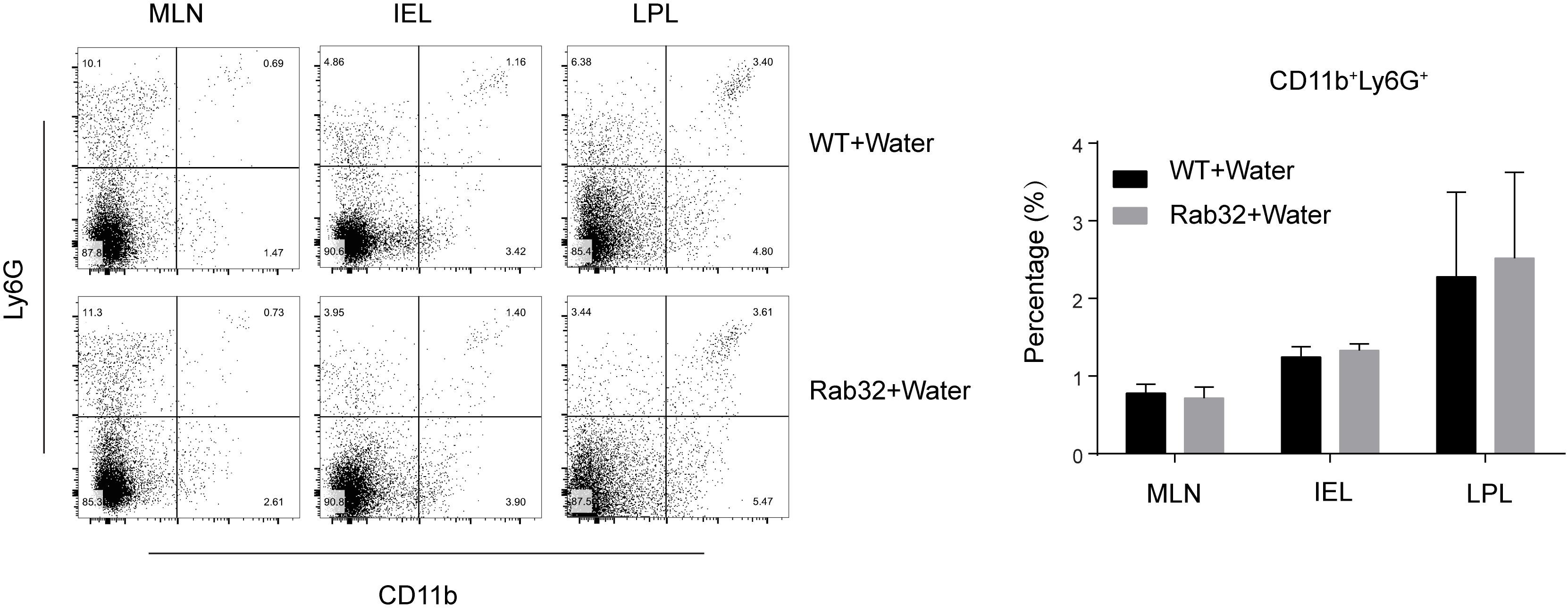

Supplement: Supplementary file 5 — Fig. S5. The proportion of neutrophils infiltrated in the colon of the WT and CD11c‐Cre+Rab32f/f mice administered water. The frequencies of neutrophils (CD11b+Ly6G+) in isolated colonic IEL, LPL and MLNs from mice in the indicated groups were determined by FACS (n = 5‐8 mice/group). All data are shown as the means ± SD. [file FEB4-8-1658-s005.tif]
