# Supplementary material for: Rab32‐related antimicrobial pathway is involved in the progression of dextran sodium sulfate‐induced colitis
Source: FEBS Open Bio. 2018 Sep 21;8(10):1658–68. doi: 10.1002/2211-5463.12514 (PMC6168699; doi:10.1002/2211-5463.12514)
Supplement: Supplementary file 8 [file FEB4-8-1658-s008.docx]

Figure S1. **Analysis of the CD11c-Cre^+^Rab32^f/f^ mice**. BMDCs and BMDMs generated from WT and CD11c-Cre^+^Rab32^f/f^ mice. (A) PCR analysis with primers amplified from exon1 and exon 3 of Rab32 with genomic from BMDCs. (B) The expression of Rab32 protein in BMDCs generated from WT and CD11c-Cre^+^Rab32^f/f^ mice were analysed by Western blot. (C) The expression of Rab32 protein in BMDMs of WT and CD11c-Cre^+^Rab32^f/f^ mice were analysed by Western blot.

Figure S2. **Histology analysis of colon tissue in the WT and CD11c-Cre^+^Rab32^f/f^ mice administered water**. HE-stained sections of the (A) caecum, (B) proximal colon and (C) distal colon from WT and CD11c-Cre^+^Rab32^f/f^ mice were microscopically examined at 40X and 200X, and histology scores were analysed for 7-8 mice each group. Scale bar: 500 μm, 100 μm. All data are shown as the means ± SD.

Figure S3. **The expression of pro-inflammatory cytokines and frequencies of T cells in the colon tissue.** (A) Total RNA was extracted from the colons tissues of untreated and DSS treat mice to analyse the expression of pro-inflammatory cytokines IL17, IFNγ and TNFα with qPCR. (B) The frequencies of CD3^+^, CD4^+^ and CD8^+^ T cells in the isolated colon from mice in the indicated groups were determined by FACS (n = 3 mice/group). All data are shown as the means ± SD.

Figure S4. **The maturation of BMDCs generated from WT and CD11c-Cre^+^Rab32^f/f^ mice after stimulated with LPS**. On Day 7 in culture, BMDCs were stimulated with 1 μg/ml LPS for 24 h. (A) The surface marker MHC II molecule, CD80 and CD86 were analysed by FACS. (B) Proportions of MHC II^hi^, CD80^+^, and CD86^+^ BMDCs were calculated. The experiments were repeated 3 times. All data are shown as the means ± SD.

Figure S5. **The proportion of neutrophils infiltrated in the colon of the WT and CD11c-Cre^+^Rab32^f/f^ mice administered water.** The frequencies of neutrophils (CD11b^+^Ly6G^+^) in isolated colonic IEL, LPL and MLNs from mice in the indicated groups were determined by FACS (n = 5-8 mice/group). All data are shown as the means ± SD.
